# Genes on a Wire: The Nucleoid-Associated Protein HU Insulates Transcription Units in *Escherichia coli*

**DOI:** 10.1038/srep31512

**Published:** 2016-08-22

**Authors:** Michael Berger, Veneta Gerganova, Petya Berger, Radu Rapiteanu, Viktoras Lisicovas, Ulrich Dobrindt

**Affiliations:** 1 Institut für Hygiene, University Münster, Mendelstraße 7, 48149 Münster, Germany; 2 School of Engineering and Sciences, Jacobs University Bremen, Campus Ring 1, 28759 Bremen, Germany; 3 Interdisciplinary Center for Clinical Research (IZKF), University of Münster, Münster, Germany

## Abstract

The extent to which chromosomal gene position in prokaryotes affects local gene expression remains an open question. Several studies have shown that chromosomal re-positioning of bacterial transcription units does not alter their expression pattern, except for a general decrease in gene expression levels from chromosomal origin to terminus proximal positions, which is believed to result from gene dosage effects. Surprisingly, the question as to whether this chromosomal context independence is a *cis* encoded property of a bacterial transcription unit, or if position independence is a property conferred by factors acting in *trans*, has not been addressed so far. For this purpose, we established a genetic test system assessing the chromosomal positioning effects by means of identical promoter-fluorescent reporter gene fusions inserted equidistantly from OriC into both chromosomal replichores of *Escherichia coli* K-12. Our investigations of the reporter activities in mutant cells lacking the conserved nucleoid associated protein HU uncovered various drastic chromosomal positional effects on gene transcription. In addition we present evidence that these positional effects are caused by transcriptional activity nearby the insertion site of our reporter modules. We therefore suggest that the nucleoid-associated protein HU is functionally insulating transcription units, most likely by constraining transcription induced DNA supercoiling.

The highly abundant nucleoid-associated protein (NAP) HU consists of two subunits HUα and HUβ and is one of the most conserved DNA binding proteins in bacteria^1^. In *Escherichia coli (E. coli*), HU was shown to play important mechanistic roles in very basic cellular functions, such as initiation of replication, chromosome partitioning and organization^2^,^3^. HU compacts DNA by constraining toroidal DNA supercoils which in contrast to the plectonemic supercoils stabilized by the global repressor H-NS do not impede transcription^4^,^5^,^6^. We showed earlier that *hupA*/*B* mutants do not form transcription foci. Transcription foci are considered to be spatially confined accumulations of RNA polymerase (RNAP) at ribosomal RNA (rRNA) operons that are characteristic for rapidly growing *E. coli* cells^7^,^8^. In addition, we could show that the global transcript pattern of *hupA*/*B* mutants displays a corresponding, chromosomal position dependent asymmetry. Genes that are up regulated in comparison to wild type cells are clustered in the rrn domain, a chromosomal region that comprises OriC and is delimited by the last rRNA operons on both arms of the *E. coli* chromosome^8^. In contrast to RNAP, HU is known to be evenly distributed throughout the chromosome of rapidly growing *E. coli* cells^8^,^9^. To explain the observed genome-wide transcription asymmetry we therefore proposed an indirect mechanism that is based on the supercoil constraining properties of HU. According to this model HU forms metastable nucleoprotein complexes by constraining the supercoils generated by transcription of the exceptionally strongly transcribed rRNA operons. In wild type cells, these metastable structures are sufficient to maintain the integrity of transcription foci and function as a “topological sink”. These HU dependent structures act as insulators of rRNA transcription units by preventing the diffusion of negative supercoiling upstream and positive supercoiling downstream of the transcribing polymerases^8^,^10^,^11^,^12^,^13^. In the absence of HU, the accumulation of negative supercoils upstream and positive supercoils downstream of active rRNA operons organizes the whole *E. coli* chromosome into a “twin-supercoil domain structure”. According to this model, the observed asymmetry of the global transcript pattern in *hupA*/*B* mutants simply results from domain-dependent differences in the superhelical tension available for the untwisting of the DNA template.

The *E. coli* chromosome in rapidly growing *hupA*/*B* mutants is apparently separated into two domains, which in turn results in differential expression of the genes within these domains. However, so far there is no evidence for a regulatory impact of the architecturally distinct nucleoprotein complexes extending over large genomic regions^14^,^15^ or isolated topological domains with variable borders^16^,^17^,^18^ on gene expression in wild type cells. Recently a study proposed the existence of chromosomal positions which result in transcriptional silencing, the so called tsEPODs (Transcriptionally Silent Extended Protein Occupancy Domain)^19^. However, all other previous investigations done in wild type *E. coli* or *Salmonella* reported no significant effects of chromosomal position on the expression of bacterial transcription units. In these studies only artefacts resulting from the absence of insulating terminators in the reporter systems and simple copy number effects that were observed when comparing the expression of identical promoter-reporter gene fusions in origin-proximal and origin-distal positions were detected^20^,^21^,^22^,^23^,^24^,^25^. Surprisingly, the question whether the observed chromosomal context independence is an in *cis* encoded property of a bacterial transcription unit or perhaps requires additional, in *trans* encoded factors, was not addressed so far. As we observed that HU obviously plays an important role in insulating physically linked genes from the impact of active rRNA operons, we were interested to know whether HU serves a similar function in organization of the active bacterial transcription units in general. For this purpose, we positioned *hns* and *dps* promoter-reporter gene modules (P*hns* and P*dps* modules) symmetrically in the *E. coli* chromosome and investigated module activity as a function of chromosomal position in cells lacking HU. Except for one H-NS dependent fusion artefact caused by the P*hns* module in a terminus position, both modules display the previously reported position independence in wild type cells. We also tested the P*dps* module in a previously described tsEPOD position (*yqe*^19^) and did not observe any effect on reporter gene expression in wild type cells. In contrast, the activity of both modules is affected by chromosomal position in *hupA*/*B* mutant cells to varying extents. In addition, we show that the supercoiling sensitive *fis* promoter is sensitive to its position with respect to a ribosomal RNA operon and that the P*dps* module is sensitive to upstream transcriptional activity in cells lacking HU. We thus infer that the context independence of bacterial transcription units is not a *cis* encoded property, but requires at least one factor acting *in trans*, namely, the NAP HU.

## Results

### The reporter modules

#### Construction of the reporter modules

**Figure 1:**
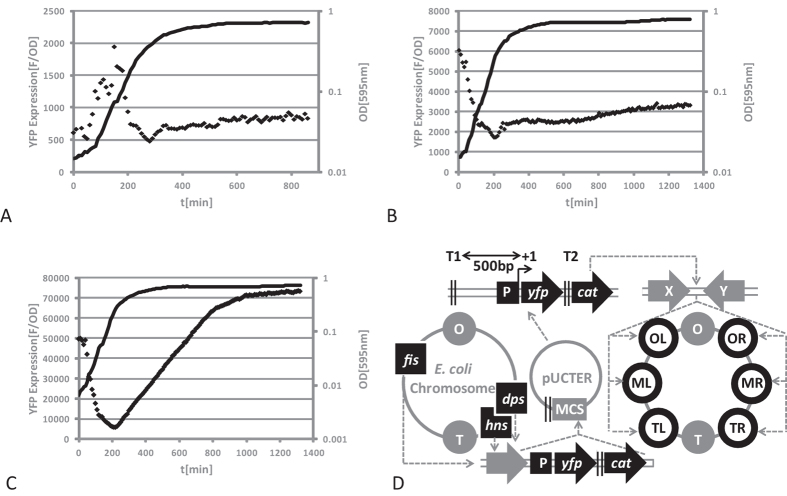
Growth phase dependent expression of different chromosomal NAP promoter-*yfp* fusion constructs and schematic overview for the construction of reporter strains used in this study. (**A**) *fis* promoter dependent YFP expression; (**B**) *hns* promoter dependent YFP expression; (**C**) *dps* promoter dependent YFP expression. The YFP expression patterns are clearly distinct and are reflecting the known growth phase dependent expression patterns of Fis, H-NS and Dps. (**D**) Flowchart showing the construction procedure of the reporter strains used in this study. O: origin of replication; OL: origin-proximal position, left replichore; OR: origin-proximal position, right replichore; ML: middle position, left replichore; MR: middle position, right replichore; T: terminus of replication; TL: terminus-proximal position, left replichore; TR, terminus-proximal position, right replichore.

Our first aim was to construct a functional reporter system that permits the measurement of chromosomal promoter activity in real time. We created a set of chromosomal transcriptional NAP promoter-*yfp* (yellow fluorescent protein) fusions by precisely replacing the open reading frames (ORFs) of *fis, dps* and *hns* genes by *yfp*. We measured YFP production for each construct in real time and the reporter gene expression (F/OD) was determined by normalizing fluorescence signal (F) to optical density (OD) for each time point as described previously^26^. Figure 1A–C shows the YFP production curve for the reporter strains over time. All the chromosomal promoter-reporter gene fusions reproduced the previously established NAP expression patterns^27^,^28^,^29^,^30^. Consistent with previous observations the *fis::yfp* expression sharply increased at the onset of exponential growth phase and YFP fluorescence declined together with cell division (Fig. 1A), *hns::yfp* was expressed constitutively with a slight increase towards the onset of stationary phase (Fig. 1B), whereas *dps::yfp* expression was activated on transition to stationary phase (Fig. 1C). After we confirmed that the YFP production from the chromosomal promoter fusions was reflecting the corresponding NAP expression patterns, we PCR-amplified the reporter modules from chromosomal DNA and cloned the reporter constructs into a plasmid (Fig. 1D). Afterwards we integrated the P*hns* and the P*dps* modules between convergent ORFs in six symmetrical chromosomal positions denoted OL (Origin-proximal position, left replichore), OR (Origin-proximal position, right replichore), ML (Middle position, left replichore), MR (Middle position, right replichore), TL (Terminus-proximal position, left replichore) and TR (Terminus-proximal position, right replichore) and the supercoiling dependent P*fis* module in ML and MLup (upstream *rrnG*) in *E. coli* K-12 strain CSH50 using the lambda RedE/T recombination system. The modules were flanked on both sides by strong *rrnB* terminators (Figs 1D and S1), insulating the promoter-reporter gene fusion from upstream transcription. For simplicity, the promoter module in chromosomal position OL is referred to as P*gene* module OL and so on.

#### Impact of reporter gene expression on physiology and technical analysis

Since the P*dps* module produced the highest amounts of YFP when compared to P*fis* and P*hns* module (compare Fig. 1C to 1A,B), we used the P*dps* module for an additional detailed technical analysis, including the response of the plasmid construct to known regulators (Figure S2A) and the impact of reporter gene expression on growth characteristics of the different reporter strains (Figure S2B). We also confirmed that the normalized YFP accumulation as measured by our reporter constructs correlates well with the actual YFP synthesis rates due to the individual promoter activities of the different modules as determined according to De Jong *et al*.^31^ (Figure S3). To obtain a comprehensive picture of the behaviour of the transplanted P*dps* modules in wild type cells we also analysed the growth phase dependent pattern of YFP expression in different growth media. Figure S2C,D show the growth curves of the six reporter strains (coloured lines), of the strain with *yfp* fused to the *dps* promoter in its native context (black line) and the wild type strain without insertion (grey line), as well as the corresponding patterns of YFP expression (diamonds) from the *dps* promoter constructs in M9 minimal medium (M9) and M9 minimal medium supplemented with casamino acids (M9 casamino acids). As already observed for the module on plasmids (Figure S2B), the growth rate and final cell density of the reporter strains did not deviate significantly from the K-12 wild type strain CSH50 (Figure S2C,D, compare coloured growth curves to the grey line). In addition, we also tested the activity of the P*dps* module in a so-called tsEPOD position (*yqe* position), for which transcriptional silencing was recently reported by Bryant *et al*.^19^. We did not observe any silencing effect on our reporter module in *E. coli* K-12 strains CSH50 and MG1655 (Figure S4A, compare black diamonds with coloured diamonds; Figure S4B). Thus the low activity of a *lac* promoter construct reported in this position might have been specific for the construct used in that study.

### Growth phase dependent expression of the P*dps* modules

**Figure 2:**
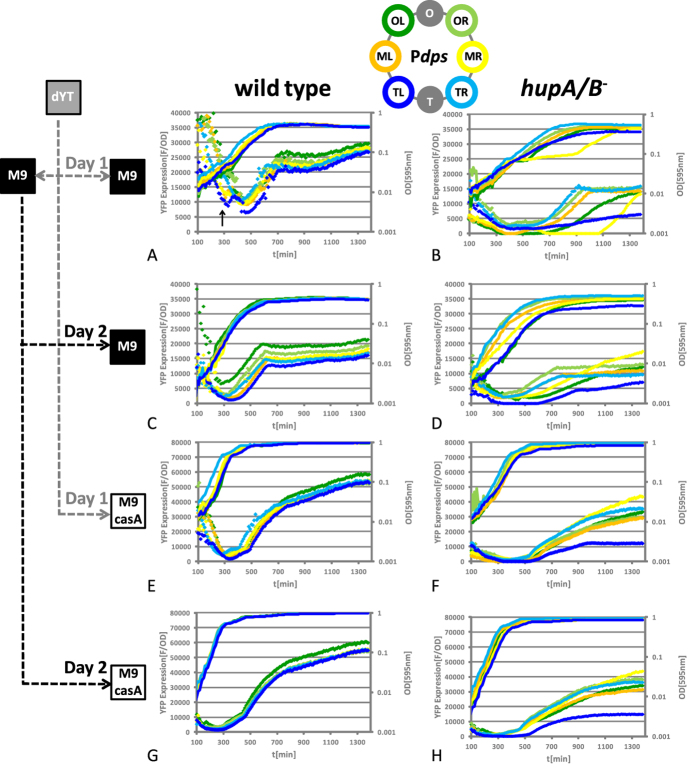
P*dps* module dependent YFP expression in wild type and *hupA*/*B* mutant. Shown are the growth curves (lines) and corresponding YFP expression patterns (diamonds) of the strains containing the P*dps* modules in six different chromosomal positions (a graphical representation of the positions and color code for the modules is shown above the graphs; the experimental setup is shown as flow chart on the left). (**A**,**E**) P*dps* module dependent YFP expression in wild type cells using dYT starter culture (Day 1). The medium switch causes an intermediate lag phase in M9 that is accompanied by an activation of the P*dps* modules (**A**; black arrow). (**C**,**G**) P*dps* module dependent YFP expression in wild type cells using M9 as starter culture (Day 2). The intermediate lag phase in M9 is absent and the growth of the strains becomes more synchronized (compare lines in **A**,**E** with **C**,**G**). The overall pattern of P*dps* module dependent YFP expression is very similar and stable in wild type cells. (**B**,**F**) P*dps* module dependent YFP expression patterns in *hupA*/*B* mutant cells using dYT starter culture (Day 1) and M9 as starter culture (Day 2; **D**,**H**), showing no detectable copy number effects and various positional effects.

Next, we constructed isogenic *hupA*/*B* mutants of the P*dps* reporter strains by P1 transduction. We analysed the growth phase dependent expression of the transplanted P*dps* modules in wild type and *hupA*/*B* mutants in 48 h experiments outlined in Fig. 2. The first day, the microtiter plate containing M9 and M9 casamino acids was directly inoculated from a dYT starter culture. The same dYT starter culture was used to inoculate an M9 starter culture, which was used to inoculate the microtiter plate on the second day.

#### Wild type

In both growth media and independent of the starter culture, P*dps* was induced at the transition to stationary phase (note the reduction of F/OD values during logarithmic growth phase; diamonds in Fig. 2A,C,E,G). Interestingly, P*dps* was also transiently activated at the onset of an intermittent lag phase when a dYT starter culture was used for inoculation in M9 (Fig. 2A, black arrow; see also Figure S2C). This intermediate lag phase was most likely due to the requirement for activating the amino acid biosynthesis pathways, as it was absent both in M9 casamino acids (Fig. 2E,G) and also when the cells were inoculated from a M9 starter culture (Fig. 2C). No strong gene copy number effects on YFP expression were observed, which was expected as P*dps* was activated when the cells stop to divide (compare the YFP expression curves to growth curves). However, YFP production showed dependence on the growth medium, with YFP expression being 2–3 times lower in M9 than in M9 casamino acids (compare YFP expression values in Fig. 2A,C with Fig. 2E,G).

#### hupA/B mutant

Next we compared the activity of the modules in the *hupA*/*B* background (Fig. 2B,D,F,H). Whereas the chromosomal insertion of the reporter modules did not show any significant effect on the growth of wild type cells, it compromised the normal growth behaviour in the mutant in chromosomal positions MR and TL (compare Fig. 2B to Fig. 2A). The reporter module MR caused an extremely prolonged intermediate lag phase, whereas the module TL resulted in a retarded growth with a slightly reduced final cell density (Fig. 2B, yellow and blue lines, respectively). In both cases the negative effect of the module on cell growth could not be attributed to an elevated expression of the reporter gene (yellow and blue diamonds). Interestingly the observed growth defect caused by the module MR was conditional, as it was absent when M9 was used as a starter culture on the second day of the experiment (Fig. 2D) and in M9 casamino acids (Fig. 2F,H), suggesting that the activation of the module MR at the onset of the intermediate lag phase caused this growth defect. When the cells were inoculated from a M9 starter culture on the second day of the experiment, the growth of the reporter strains was more synchronized (compare slopes of the lines in Fig. 2D with 2B and 2H with 2F). Under these conditions, MR produced the highest amounts of YFP in M9 and in M9 casamino acids, whereas the module TL still produced the lowest amount (Fig. 2B,D,F,H). We could exclude any role of secondary mutations as explanation for the low expression of the module TL because P1 transduction of this allele into wild type cells restored its normal expression strength (Figure S4A, compare purple and blue diamonds). In order to clarify the cause of the observed low fluorescence signal produced by the modules MR and TL after the switch from complex to minimal medium, we determined the distribution of fluorescence intensities within stationary phase cell populations by FACS analysis. As shown in Figure S5, the fluorescence signals were normally distributed in both wild type and *hupA*/*B* mutant cells. We therefore concluded that the low fluorescence signals produced by the modules MR and TL in *hupA*/*B* mutants did not result from heterogeneity of the bacterial population with respect to YFP expression, but rather from an overall weak activity of the promoter in these positions.

### OxyR mediated response of the P*dps* modules

**Figure 3:**
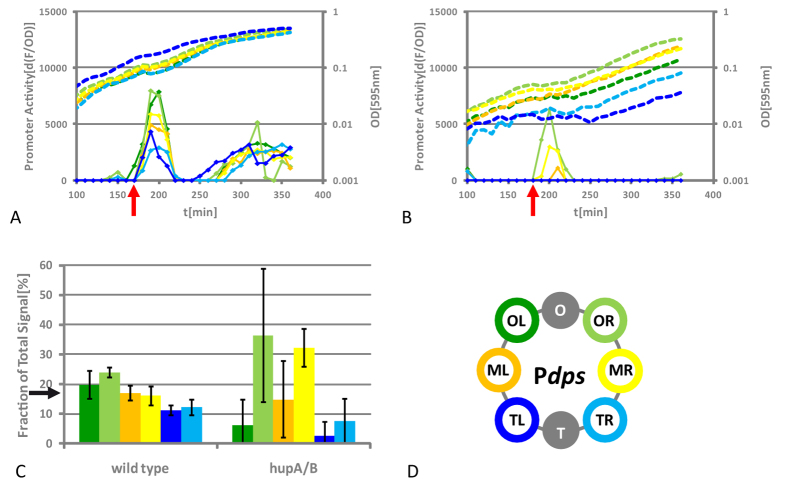
P*dps* module activity after hydrogen peroxide challenge. Shown are the promoter activities of the P*dps* modules as increase of normalized fluorescence over time (diamonds and lines) and corresponding growth curves (dashed lines). Red arrows indicate time point of H_2_O_2_ addition. (**A**) In wild type cells, all P*dps* modules are producing YFP for about 40 min after H_2_O_2_ addition. Afterwards the P*dps* is switched off until it is reactivated by growth phase control. As expected, the copy number effects are more pronounced in this experiment (compare module position and YFP production). (**B**) In *hupA*/*B* mutants, only a subset of the P*dps* modules produced detectable fluorescence in this experiment, no copy number effects can be observed. (**C**) P*dps* module response as fraction of total signal in 5 independent experiments. The expected value is approximately 17% (100/6), if all positions contribute equally to the total response (black arrow). In contrast to wild type, the response of the modules is strongly fluctuating in the *hupA*/*B* mutant with no indication of copy number effects. (**D**) Schematic representation of P*dps* module positions in the *E. coli* chromosome.

Next, we investigated the OxyR-mediated response of the P*dps* modules to oxygen radicals (H_2_O_2_ treatment) in exponential growth phase in wild type and *hupA*/*B* mutant (Fig. 3).

#### Wild type

Figure 3A depicts the growth curves (dashed lines) of the reporter strains together with their *dps* promoter activity as increase of fluorescence over time. All reporter modules were induced on addition of sub-lethal concentrations of H_2_O_2_ (red arrow) for about 40 min, after which the increase of fluorescence stopped until the promoter was re-activated on the transition to stationary phase. As expected, the copy number effects became more noticeable during rapid growth, as the modules in the origin-proximal positions which exhibited higher activity when compared to origin-distal positions. As judged by the small variations in between the biological replicates, the relative response of the modules to oxidative stress in wild type cells was very stable (Fig. 3C).

#### hupA/B mutant

We then compared the reporter response to H_2_O_2_-stress in logarithmic growth phase in *hupA*/*B* mutants (Fig. 3B). In this experiment, only a subset of modules showed a measurable response in (OR, MR and ML). The variable extend of transcriptional activity of different modules was characteristic for the *hupA*/*B* background: The response of the individual modules in cells lacking HU varied strongly in-between the replicates, without any indication of copy number effects and P*dps* module TL was again among the weakest expressed modules (Fig. 3C). Whereas the decrease in YFP production from origin-proximal to terminus-proximal positions was clearly observable in wild type, no such pattern occured in *hupA*/*B* mutants (compare for example YFP production in OL and MR).

### Comparison of the P*hns* modules in *E. coli* K-12 wild type cells and isogenic *hupA*/*B* mutants

**Figure 4:**
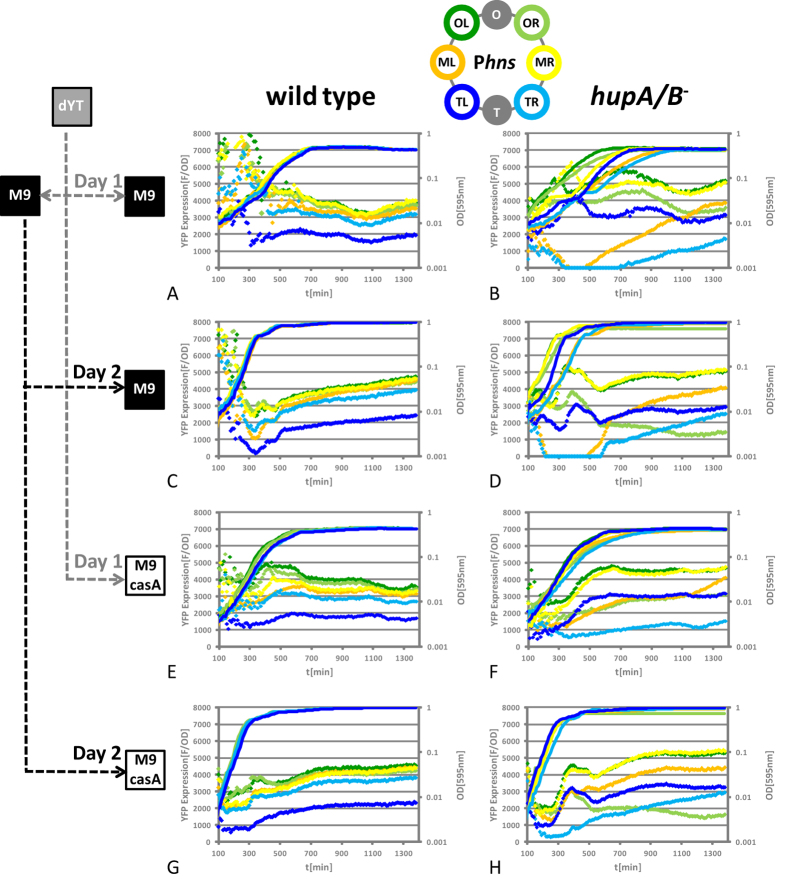
P*hns* module dependent YFP expression in wild type and *hupA*/*B* mutant. Shown are the growth curves (lines) and corresponding YFP expression patterns (diamonds) of the strains containing the P*hns* modules in six different chromosomal positions (a graphical representation of the positions and color code for the modules is shown above the graphs; the experimental setup is shown as flow chart on the left). (**A**,**E**) P*hns* module dependent YFP expression in wild type cells using dYT starter culture (Day 1). (**C**,**G**) P*hns* module dependent YFP expression in wild type cells using M9 as starter culture (Day 2) Except for TL, the overall pattern of P*hns* module dependent YFP expression is very similar and less dependent on the growth medium than the P*dps* modules in wild type cells. (**B**,**F**) P*hns* module dependent YFP expression patterns in *hupA*/*B* mutant cells using dYT starter culture (Day 1) and M9 as starter culture (Day 2; **D**,**H**), showing no detectable copy number effects and various positional effects.

We also addressed the question, as to whether the observed sensitivity to chromosomal position was a general characteristic of the *hupA*/*B* mutant. For this purpose we analysed the reporter strains with chromosomally integrated *Phns* reporter modules in the same manner as described above for the P*dps* module containing strain sets. Figure 4 shows a typical result of a 48 h experiment.

#### Wild type

In comparison to the P*dps* modules, the P*hns* modules in the wild type maintained the intracellular YFP concentrations at relatively stable levels during growth (Fig. 4A,C,E,G). The intracellular YFP levels were less dependent on the growth medium when comparing M9 and M9 casamino acids (compare Fig. 4A,C with E,G), whereas overnight incubation in complex medium apparently resulted in higher activity of P*hns* (compare diamonds up to ~300 min in Fig. 4A with C,E with G). When M9 was used as starter culture on the second day of the experiment, YFP levels stayed relatively constant throughout the experiment, with a slight reduction in early log phase and a subsequent increase toward the initial YFP levels that started in mid exponential phase both in M9 and M9 casamino acids (Fig. 4C,G). As expected for a rather constitutive promoter, copy number effects were noticeable for the P*hns* modules in exponential growth phase, where YFP levels decreased with the distance of the module position from the origin of replication. Whereas the overall differences in YFP production from the P*hns* modules with respect to promoter kinetics and final YFP amounts were relatively low in wild type cells, the module in TL produced less YFP than all the other modules, independent of growth medium and starter culture (Fig. 4A,C,E,G blue diamonds). The low amount of YFP that was produced by P*hns* module TL was unlikely to result from copy number effects, as the symmetrical P*hns* module TR produced almost twice as much YFP. However, in cells lacking H-NS the activity of the P*hns* module TL was restored and YFP levels were similar to the rest of the modules (Figure S6), which suggested that the observed low activity was caused by an H-NS dependent repression mechanism that was specific for the P*hns* module TL. The reduced activity of the module was not therefore a consequence of local interactions with the promoter region, but rather of the artificial fusion of the reporter module with the chromosomal backbone in this position.

#### hupA/B mutant

Figure 4B,D,F,H show the YFP expression patterns of the P*hns* modules in the *hupA*/*B* mutant background. In contrast to the P*dps* modules, the P*hns* modules were not generally weaker expressed in the *hupA*/*B* mutant. Dependent on the position, the modules were either weaker (e.g. TR) or stronger (e.g. MR) expressed than in the wild type (Fig. 4). In contrast to the P*dps* module MR the P*hns* module MR did not cause a conditional growth defect, which indicated that the observed growth defect was specific for the *dps* promoter. As for the P*dps* modules, both kinetics and expression strength were affected by the chromosomal position in cells lacking HU and the different YFP amounts produced by the module could not be explained by copy number effects. Note that e.g. the module OR produced less YFP than TL and TR when M9 was used as a starter culture (Fig. 4D,H). Independent of the starter culture the overall pattern of YFP expression for each module was very similar within a 48 h experiment (compare diamonds in Fig. 4B,D,F,H).

### Chromosomal position dependent module activity in the *hupA*/*B* mutant: A possible mechanistic role of transcription induced DNA supercoiling

#### The fis promoter: Ribosomal RNA operon G constitutes a domain border in hupA/B mutants

**Figure 5:**
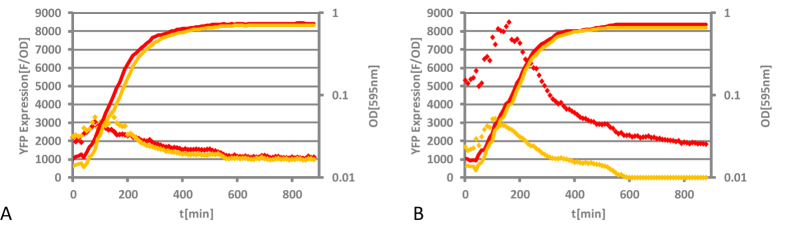
P*fis* module dependent YFP expression in wild type (**A**) and *hupA*/*B* mutant (**B**). Shown are the growth curves (lines) and corresponding YFP expression patterns (diamonds) of the strains containing the P*fis* modules in MLup (red) and ML (orange). The P*fis* modules ML and MLup produce identical amounts of YFP in wild type cells (compare red and orange diamonds in **A**). In the *hupA*/*B* mutant the P*fis* module MLup produces 2–3 fold more YFP than P*fis* module ML (compare red and orange diamonds in B).

We demonstrated earlier that the *hupA*/*B* mutation resulted in a spatial asymmetry of global genomic transcription in exponential growth phase. We suggested that this asymmetry most likely resulted from elevated levels of free negative supercoiling that were caused by the strong transcription of ribosomal operons and the unobstructed access of DNA gyrase to binding sites enriched in the rrn domain^8^. Here we addressed the question as to whether the ribosomal RNA operon G (*rrnG*) constitutes the expected domain border for the supercoiling sensitive P*fis* module in the *hupA*/*B* mutant. Position ML was localized downstream of *rrnG*, whereas MLup was localized directly upstream of this operon and therefore within the rrn macrodomain^8^. We found that the P*fis* module ML produced similar YFP amounts in the *hupA*/*B* mutant when compared to wild type cells in logarithmic growth phase (compare orange diamonds in Fig. 5A,B; see also Figure S7A,B). This was not surprising, since the level of constrained supercoiling is known to be substantially reduced in *hupA*/*B* mutant^32^ whereas we have shown that the unconstrained superhelical density is at least as high as in wild type cells^8^. Most importantly, in the *hupA*/*B* mutant the supercoiling sensitive P*fis* module showed 2–3 times higher expression values in MLup than in ML, as expected (Fig. 5B), which is indeed resulting from differences in YFP synthesis rates in the mutant (Figure S7B), but not from an overall distinct expression pattern (Figure S7D). In contrast to the *hupA*/*B* mutants, the actual chromosomal position of the P*fis* module was not affecting the reporter gene expression in wild type cells (Fig. 5A). The fact that the supercoiling sensitive P*fis* reporter system detects *rrnG* as a border of the rrn domain lends additional support to our hypothesis that a perturbed topological homeostasis is resulting in transcriptional interference in the *hupA*/*B* mutant^8^.

#### Promoters are sensitive to upstream transcriptional activity in the hupA/B mutant

Our observations with all three reporter modules indicated that not only the strong transcriptional activity of ribosomal RNA operons, but the transcription of any gene may interfere with the transcription of adjacent genes in the *hupA*/*B* mutant.

**Figure 6:**
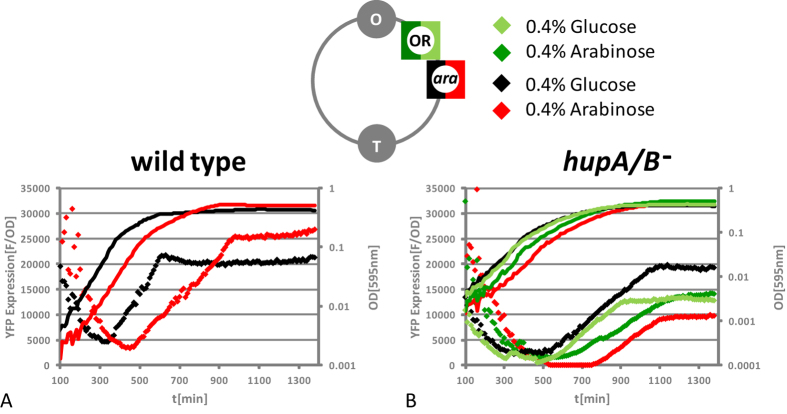
Upstream gene expression is repressing the P*dps* module *ara* in the *hupA*/*B* mutant. Shown is the YFP production of the P*dps* modules OR and *ara* in M9 supplemented with glucose and M9 supplemented with arabinose. Only the P*dps* module *ara* is repressed in M9 supplemented with arabinose (compare red with black diamonds).

We tested this hypothesis by inserting the P*dps* module downstream of the *araBAD* operon (denoted P*dps* module *ara*). The results are shown in Fig. 6. When the cells were grown in M9, the *araBAD* operon was inactive and YFP levels produced by the P*dps* module *ara* in the *hupA*/*B* mutant reached wild type levels (compare black diamonds in Fig. 6A,B). However, when the cells were grown in M9 medium supplemented with arabinose (M9 Ara), YFP levels dropped below detection limit between 500 min–700 min and the P*dps* module *ara* produced only in the *hupA*/*B* mutant approximately half as much YFP in comparison to M9 (Fig. 6, compare red diamonds with black diamonds). As we noticed that YFP expression from the P*dps* modules were sensitive to the growth medium both in wild type and *hupA*/*B* mutant (Fig. 2), low YFP expression in M9 Ara might have been explained by an arabinose specific effect on the *dps* promoter in the *hupA*/*B* mutant (in wild type cells the module produces more YFP in M9 Ara, Fig. 6A). However, even though the kinetics of the proximal P*dps* module OR that we used as a control were apparently affected by the carbon source, the final YFP amounts produced in M9 Ara were similar to the YFP amounts produced in M9 (Fig. 6B, compare dark green and bright green diamonds). This arabinose dependent inhibition of the P*dps* module *ara* in *hupA*/*B* mutants was highly reproducible and, as expected, not observed in wild type cells (Figure S8A,B).

## Discussion

This study provides for the first time evidence for the hypothesis that the functional context independence of prokaryotic transcription units is not an *in cis* encoded property. We comprehensively investigated both the expression strength and dynamics of the *E. coli dps, hns* and *fis* promoters fused to a fluorescent reporter in different chromosomal positions along the OriC-Ter axis in *E. coli* K-12. As reported previously for other reporter constructs, the promoters were insensitive to the chromosomal context in wild type cells, except for the P*hns* module TL. However, we observed strong chromosomal position dependent differences in *dps, hns* and *fis* promoter activity in cells lacking the conserved NAP HU (Figs 2, 4 and 5). This result might not be surprising for a supercoiling sensitive promoter like the *fis* promoter. Fis expression is activated at the onset of logarithmic growth and we already reported the existence of two topologically distinct chromosomal domains for this growth phase in the *hupA*/*B* mutant^8^. However, as H-NS is a rather constitutively expressed DNA architectural protein and Dps is a maintenance protein that is protecting the chromosomal DNA in hazardous environments, the *hns* and *dps* promoter regulation is very different from that of the *fis* promoter^32^,^33^,^34^,^35^.

All known regulator binding sites implicated in the regulation of the *fis, hns* and *dps* genes are within the confines of the sequence used for the construction of our reporters^35^,^36^,^37^. Except for the H-NS dependent fusion artefact for the P*hns* module TL, our reporters behaved uniformly both in terms of strength and kinetics throughout the different growth phases and appeared to be independent of chromosomal position in *E. coli* K-12 wild type cells (Figs 2 and 4). All the P*dps* modules also coherently responded to oxidative stress with origin-proximal positions showing a stronger response, than the terminus-proximal ones due to the impact of copy number effects during exponential growth (Figs 3B and 4B)^20^,^21^,^22^. Therefore, we infer that the modules used fulfil the requirements of reliable reporters, insulated from surrounding direct transcriptional influences and largely independent of the chromosomal context in wild type cells within our experimental setup. It should be also noted here that *gfp* – as well as the commonly used reporter gene *lacZ* - were shown to be bound by H-NS^38^,^39^. Therefore it is likely that the *gfp* derivative *yfp* is bound by the NAP H-NS as well. However, the generally uniform behaviour of the modules in wild type cells showed that a potential local binding of H-NS to *yfp* did not result in differential expression of the modules. Moreover, the exceptionally low activity of the P*hns* module, but not the P*dps* module TL in wild type cells indicates that not the reporter gene *per se*, but the combination of a specific site of integration with a specific construct is more likely to cause H-NS dependent silencing artefacts (Fig. 4A,C,E,G, blue diamonds). This H-NS dependent artefact in particular might be caused by the combination of H-NS binding sites in the *hns* promoter and the exceptionally GC-poor upstream region resulting from the integration of the construct in this position: 1000 bp upstream of the transcription start site have a GC content of 41.6% for the P*hns* (for the P*dps* in the same position the GC content is 44.4%). A similar explanation was also favoured by Brambilla and co-workers who also analysed the P*hns* modules in wild type bacteria^40^. H-NS was shown to transcriptionally repress horizontally acquired DNA with low GC content in pathogenic *E. coli*, most likely by polymerization from high affinity nucleation sites^41^,^42^. This repression mechanism is known as xenogenic silencing and it is an interesting observation that such a repressed region can obviously also be artificially constructed in *E. coli* K-12. It should be also noted here that others were also describing a mild effect of position OL on the P*hns* module under very specific growth conditions in wild type cells^40^. We did not observe this within our experimental setup, but the slightly higher expression of the P*hns* module OL than OR in the *hns* mutant background might indicate a similar, but less pronounced fusion dependent H-NS repression at this position as well (Figure S6). In contrast to all previous investigations on positional effects on gene expression that were done in *E. coli*, as well as in *Salmonella*^20^,^21^,^22^,^23^,^24^,^25^, positional effects on the *lac* promoter were recently reported for wild type *E. coli*^19^. However, we did not observe any effect on the activity of our module, suggesting that the reported absence of reporter gene expression in this position could have been specific for the construct used in that study (Figure S4A; note that the module was also properly expressed in *E. coli* MG1655, Figure S4B). Another possible source for strong variations in reporter gene expression might have been a heterogeneous expression of GFP within the bacterial populations^23^ that could have resulted from a generally low expression of the specific *gfp* variant^43^. This possibility could be easily excluded, as tsEPODs should also have silencing effects on the kanamycin resistance gene that was present in the initial construct of Bryant and colleagues, which in turn should be reflected by a substantial reduction in the minimal inhibitory concentration of kanamycin^19^. However, until it is clarified if tsEPODs have silencing effects on other promoters than P*lac*, we have to infer here that positional effects on gene expression in *E. coli* K-12 wild type cells are caused by (i) relative promoter copy number, (ii) potentially arising artefacts resulting from upstream read-through in the absence of efficient terminators, or (iii) H-NS dependent fusion artefacts.

In contrast to the situation in the wild type, we observed several positional effects on our reporter modules in *hupA*/*B* mutants. First, both the P*hns* and P*dps* modules exhibited chromosomal position dependent expression patterns in *hupA*/*B* mutants that could not be explained by copy number effects. Within the *hupA*/*B* background, the P*hns* modules showed differences in activity that are at least in the same order of magnitude as the H-NS dependent repression of the P*hns* module TL in wild type cells (Fig. 4). Notably, if only the information about the behaviour of one of the modules in wild type and *hupA*/*B* mutants would be available, contradicting conclusions about the mode of action of HU on the *hns* promoter would have been made: HU is an activator in TR, but a repressor in MR (Fig. 4, compare yellow and bright blue diamonds in wild type and *hupA*/*B* mutant). This indicates that the mode of action of HU is very unlikely explained by local interactions with the promoter region. In addition, we observed compromised expression patterns of P*dps* modules that were partially associated with growth defects and strong fluctuations of the module response in between biological replicates (Fig. 3B). Such variations could be partially also observed for the P*hns* modules (compare bright green and dark green lines in Fig. 4F,H with Figure S9). Second, the supercoiling sensitive P*fis* module was more active when placed upstream of *rrnG* in comparison to the insertion downstream of *rrnG* (Fig. 5). Active transcription was shown to increase negative supercoiling upstream and reduce negative supercoiling downstream of this ribosomal RNA operon also in *Salmonella*^44^, but to our knowledge a measurable effect of this local disturbance of DNA topology on gene expression in wild type cells was not reported so far. Notably, HU is conserved among bacterial species and acts as a homeostatic regulator of the chromosomal DNA superhelical density^1^,^32^. Furthermore, during exponential growth the homeostatic balance of an apparent gradient of DNA superhelicity along the OriC-Ter axis is likely impaired in cells lacking HU^8^,^45^. On binding DNA HU can constrain both negative^46^,^47^ and - at least in the case of a mutant derivative of HU - positive^48^ supercoils. The latter was shown to result in a substantial reorganization of the global transcription program of *E. coli* K-12, including the activation of silent, virulence-associated genes, resulting in dramatic phenotypic alterations such as host cell invasion and intracellular replication. Notably, these changes were also associated with an increased global supercoiling level^49^,^50^. In the complete absence of HU it is therefore conceivable that the diffusion of the free negative and positive supercoils generated upstream and downstream of the translocating transcription machinery^11^,^51^,^52^ proceeds unobstructed, explaining the higher activity of the supercoiling-sensitive P*fis* module in MLup located upstream of the *rrnG* operon compared to ML, which is located downstream (Fig. 5). The same mechanism can result in the up regulation of genes within the rrn domain in the *hupA*/*B* mutant, as we have already proposed earlier^8^.

Is it possible to explain the up regulation of genes within the rrn domain, the position dependence of promoter-reporter gene modules, as well as the observed fluctuations in the activity of individual modules in between biological replicates in the *hupA*/*B* background by the same HU dependent mechanistic principle? NAPs are integrated into a complex regulatory network and the deletion of a NAP gene therefore most likely results in an overall change in the relative intracellular concentrations of the members of this class of abundant DNA binding proteins^53^. It is therefore in general difficult to assign to a NAP like HU a singular mechanistic role in sustaining the chromosomal context independent expression of bacterial genes. It is for example possible – as already suggested by others – that in cells lacking HU the general repressing effect of the global silencing protein and HU antagonist H-NS becomes more conspicuous^54^. However, the absence of H-NS (or Fis) did not affect the expression of the P*dps and* P*hns* module to such an extent as the absence of HU (Figure S10, Figure S6). Therefore, in contrast to the NAPs Fis and H-NS, HU appears to play a mechanistically more direct role in not necessarily regulating, but organizing the active transcription units in a way that allows for independent expression of physically linked genes. HU is a DNA architectural protein and we therefore favour a mechanism that is directly based on its DNA binding properties as explanation for our observations. We already proposed such a mechanism on the global level for the genes within the rrn domain^8^. Here we present evidence for the hypothesis that supercoils generated by active transcription might also “diffuse” into proximal transcription units and thereby aberrantly repress or activate transcription at the local level as well. We tested this hypothesis by inserting the P*dps* module directly downstream of the *araBAD* operon. And indeed, we show here that the observed repression of the P*dps* module in the presence of arabinose was clearly specific for this position in *hupA*/*B* mutants (Fig. 6B, compare to P*dps* module *ara* to P*dps* module OR; Figure S8B). In wild type cells the transcription units were topologically insulated from each other by HU and thereby insensitive to the activity of the surrounding genes (Fig. 6A; Figure S8A). Thus, the cause for position dependence of transcription units, as well as for the partially observed activity fluctuations in individual promoter modules in the *hupA*/*B* mutant is most likely alternating module proximal transcriptional activity. In the absence of HU dependent constraint of DNA supercoiling these changes in transcriptional activity are then transmitted to the modules by changes in superhelical tension. In accordance with the twin-domain model of transcription that was proposed by Liu and Wang^11^, these changes in superhelical tension are then either facilitating or inhibiting the unwinding of DNA, which is essential for an efficient transcription process. In the same way the specific transcriptional activity of the modules themselves can be transmitted to nearby genes in the *hupA*/*B* mutant. This might explain the observed promoter module and position specific growth defects. Thus, functional position independence of genes within the bacterial chromosome is not an intrinsic property of the transcription units. The functional insulation of genes in *E. coli* requires at least one *in trans* encoded factor, the conserved NAP HU.

We intend to further investigate the mechanistic role of HU in the organization and insulation of active transcription units by a more detailed approach using complex reporter modules including inducible up- and downstream genes. This should help us to further quantify the impact and effective distance of module proximal transcription on reporter modules in more detail.

## Methods

### Bacterial strains and construction of reporter strains

For cloning of plasmids and sub-constructs, please see the supplementary materials and methods. All bacterial strains used in this study are listed in Table S1B, schematic representations of the exact module positions in the reporter strains are shown in Figure S1. The *E. coli* K-12 CSH50 reporter strains were constructed with the RedE/T system (Gene Bridges Heidelberg, Germany), using pMB54, pRR1 and pVGfis4 as template and the primers listed in Table S1A, as described previously^8^. The P*dps* module *ara* was initially constructed in *E. coli* K-12 MG1655, as *E. coli* K-12 CSH50 does not possess a functional *araBAD* operon. The correctness of the insertions was verified by PCR over the synthetic 5′- and 3′-junctions of the modules and chromosome. The complete promoters, including the junction to *yfp* were additionally sequenced to rule out point mutations. For the measurements, wild types and subsequently NAP mutants were constructed by P1 transduction, using the construction strain as first donor as described previously^8^. P1 transduction of the P*dps* module *ara* from *E. coli* K-12 MG1655 *Pdps ara* restored the *ara* ^+^ phenotype in CSH50 wild type and *hupA*/*B* mutant.

### Growth conditions and YFP expression measurements

All media components used in this study were purchased from Sigma-Aldrich (St. Louis, MO). For all reporter gene expression measurements, the strains were inoculated directly from glycerol stocks in 2 ml 2xYT medium and incubated over night at 37 °C/180 rpm in an Infors HT Multitron Standard incubator (Infors, Einsbach, Germany) for the first day of the experiment. The same cultures were used to inoculate the starter cultures for the second day of the experiment (M9 supplemented with 0.4% glucose). Unless stated otherwise, M9 medium was supplemented with 0.4% glucose in all experiments. To determine the effect of arabinose on the *ara* P*dps* module, M9 was supplemented with 0.4% arabinose. For the measurements of growth phase dependent YFP expression, the overnight cultures were next day inoculated 1:200 in 150 μl M9 0.4% glucose or M9 0.4% glucose supplemented with 0.4% casamino acids in black μ-clear plates with transparent bottom (Greiner Bio-One, Frickenhausen, Germany), covered with a transparent lid and sealed with parafilm. Afterwards the plate was incubated at 37 °C in a TECAN Infinite F200 instrument (TECAN, Männedorf, Switzerland) for 23 hours with orbital shaking with an amplitude of 2 mm. Optical density at 595 nm and fluorescence signal (excitation wavelength 485 nm, emission wavelength 535 nm, detector gain 50) were recorded automatically every 10 min. Optical density signal was corrected for blank and fluorescence signal was corrected for background (CSH50 wild type for all strains). Afterwards YFP expression (F/OD) was calculated by normalizing corrected fluorescence signal (F) to corrected optical density (OD) for each time point, as described previously^28^. Promoter activity was calculated by subtracting the F/OD value of the previous time point (promoter activity [d(F/OD)]_n_ = (F/OD)_n_ − (F/OD)_n−1_). For the graphic representations negative promoter activity values were set to zero. The exemplary calculation of YFP synthesis rate normalized YFP concentration of the *fis* promoter modules were done with the MatLab software according to De Jong *et al*.^31^, except for the knot selection for the spline fitting. Whereas De Jong and colleagues describe the use of a generalized cross-validation criterion (GCV) for determining the number and the placement of the knots, we used the Schoenberg Whitney conditions for knot selection. The reason for selecting this method was for speed and simplicity in the code, while sacrificing smoothness of the spline fit, in comparison to using GCV for knot selection. However, when compared to the raw data, it’s found that the Schoenberg Whitney conditions for knot selection in combination with the least-squares cubic spline are sufficient to maintain the integrity of the signal while reducing experimental noise significantly. For the repetition of the measurement the next day, 2 ml of M9 medium supplemented with 0.4% glucose were inoculated directly from the initial dYT starter culture with a microloop and incubated over night at 37 °C/180 rpm in an Infors HT Multitron Standard incubator. The next day the procedure was repeated as described above, except that the M9 starter culture was used for inoculation of the microtiter plate. Measurements of the response of the modules to reactive oxygen in logarithmic growth phase were done in the same way, except that the overnight cultures were inoculated 1:200 in 150 μl M9 0.4% glucose supplemented with 0.4% casamino acids. For the induction experiments, the detector gain of the Tecan Infinite F200 instrument was set to 70. After 170 min of incubation in the instrument, the plate was removed and 5 μl of H_2_O_2_ solution was added to a final concentration of 50 μg/ml with a multichannel pipet and the measurement was continued for additional 170 min. As the response of all P*dps* modules was essentially over after 40 min exposure to this sub lethal concentration of H_2_O_2_, YFP expression in response to H_2_O_2_ was defined as the total increase in F/OD after 40 min of treatment compared to the pretreatment F/OD value. In order to compare the response of the promoter modules in between biological replicates, the response of an individual position was normalized to the total response of all positions within each background for each experiment.

### Fluorescence activated cell sorting (FACS) analysis of fluorescence signal distribution

The reporter strains were directly inoculated from glycerol stocks in 2 ml 2xYT and grown overnight at 37 °C with shaking (150 rpm). The next day all cultures were diluted 1:200 in 10 ml M9 supplemented with 0.4% glucose in a 50 ml flask and grown at 37 °C with shaking (150 rpm). At the 23^rd^ hour all cultures were diluted 1:10 in 1X PBS, fixed in 1% PFA for 5 min at room temperature. Afterwards the bacterial cells were stained with 4′,6-diamin-2-phenylindol (DAPI) and sorted within a time interval of 2 min for each strain in a FACS machine (MoFlo, Beckman Coulter, Krefeld, Germany). On average 200 000 cells were sorted for each strain. YFP signals were recorded using a 488 nm/6 excitation filter and a 531 nm/40 emission filter. DAPI signals were recorded using a multiline UV450 nm/50 filter. Raw data were collected with the Beckman Coulter MoFlo Summit software. Images and statistics data were generated with the WinMDI2.9 software from the fcs files obtained after cell sorting.

### Software

The data were processed with Microsoft Excel and the graphic representations were done with Microsoft PowerPoint and Adobe Illustrator.

## Additional Information

**How to cite this article**: Berger, M. *et al*. Genes on a Wire: The Nucleoid-Associated Protein HU Insulates Transcription Units in *Escherichia coli.* *Sci. Rep.* **6**, 31512; doi: 10.1038/srep31512 (2016).

## Supplementary Material

Supplementary Information
