# Supplementary material for: Genes on a Wire: The Nucleoid-Associated Protein HU Insulates Transcription Units in Escherichia coli
Source: Sci Rep. 2016 Aug 22;6:31512. doi: 10.1038/srep31512 (PMC4992867; doi:10.1038/srep31512)
Supplement: Supplementary Information [file srep31512-s1.pdf]

Supplementary information

Genes on a Wire: The Nucleoid-Associated Protein HU Insulates Transcription Units in *Escherichia coli*

Michael Berger[1#*], Veneta Gerganova[2#], Petya Berger[1], Radu Rapiteanu[2], Viktoras Lisicovas[2], Ulrich Dobrindt[1, 3*]

[1]Institut für Hygiene, University of Münster, Mendelstraße 7, 48149 Münster, Germany

[2]School of Engineering and Sciences, Jacobs University Bremen, Campus Ring 1, 28759 Bremen, Germany

[3]Interdisciplinary Center for Clinical Research (IZKF), University of Münster, Münster, Germany

Supplementary Materials and Methods

Cloning of sub-constructs

Basic laboratory methods, composition of the required buffers and growth media (e.g. electrophoresis, preparation of electro competent cells) are described elsewhere (46). For all cloning procedures *E. coli* DH5α (Invitrogen, Carlsbad, CA) was used as host strain. When necessary, the QIAGEN plasmid mini kit (QIAGEN, Hilden, Germany) was used for preparations of plasmids and the QIAGEN Qiaquick PCR Purification Kit or QIAGEN Quiaquick Gel Extraction Kit was used to purify DNA from enzymatic reactions according to the manufacturers' instructions. All primers used for the clonings were purchased from Eurofins MWG Operon (Eurofins, Hamburg, Germany) and are listed in Table S1 A. Custom sequencing of plasmids and PCR products was done by Eurofins MWG operon. If not stated otherwise, Vent DNA Polymerase (New England BioLabs, NEB, Frankfurt, Germany) was used to PCR-amplify DNA for cloning and Taq DNA Polymerase (NEB) was used for screening PCRs for the identification of correct clones. All Restriction Enzymes used in this study were purchased from NEB, T4 DNA Ligase was purchased from Fermentas (Fermentas life technologies, Darmstadt, Germany). The relevant parts of all constructs were sequenced after standard screens by PCR and restriction digestion of isolated plasmids. Construction of pBADr-*yfp*. In order to improve YFP expression in *E. coli*, *yfp* was PCR-amplified from pLAU53 (47) with primer rplL_for (containing a 5´overhang with a NcoI site followed by codons coding for Met-Val and the first seven codons of the abundant *E. coli* protein RplL; Patent PCT/DE2011/000206 PCT) and c/yfp_rev. The PCR product was cut with NcoI and cloned into the NcoI/SmaI sites of pBAD24. Sequencing of *yfp* revealed two point mutations that resulted in amino acid substitutions Asp 138->Asn and Asp 182->Gly that did not affect fluorescence. Construction of sub construct pUCr-*yfp*-cat.*Yfp* and *rrnB* Terminator were amplified from pBADr-*yfp* with primers rplL_for and MBPD79 (containing an NsiI site) and digested with NsiI. The *cat* gene was PCR-amplified with primers MBPD80 (containing a PstI site) and MBPD81 (containing a KpnI site) and digested with PstI and KpnI. Both processed PCR-fragments were cloned into a HindIII, Mung Bean nuclease and subsequently KpnI digested pUC18 backbone. Construction of pUCTER. The *rrnB* Terminator was PCR amplified from pBAD24 with primers MBPD136 (containing a HindIII site) and MBPD137 (containing a MCS and a BamHI site), the PCR product was digested by HindIII and BamHI and cloned into a HindIII/BamHI digested pUC18 backbone.

Cloning of pMB54

Construction of a chromosomal *Pdps-yfp* fusion in *E. coli* CSH50. The *yfp-cat* containing DNA fragment was PCR-amplified from pUCr-yfp cat with Phusion DNA Polymerase (Finnzymes, life technologies, Darmstadt, Germany) and Primers MBPD132/MBPD133 and integrated into the CSH50 chromosome with RedE/T recombination (Gene Bridges, Heidelberg, Germany) as described previously (8). Homologous recombination results in of the chromosome with this PCR product results in a precise replacement of the start codon of *dps* with the start codon of *yfp*. The synthetic *dps* promoter-*yfp* junction was amplified from chromosomal DNA by PCR and the resulting PCR product was sequenced to verify the correctness of the transcriptional fusion. The resulting strain was hereafter named CSH50 P*dps-yfp*. A P*dps* (-500)-*yfp-cat* containing DNA fragment was PCR-amplified from chromosomal DNA of CSH50 P*dps-yfp* with Phusion DNA Polymerase and Primers MBPD88/MBPD156, digested with KpnI and cloned into a NruI/KpnI digested pUCTER backbone. The plasmid was isolated and the correctness of the synthetic P*dps-yfp* junction was verified by sequencing. The plasmid was hereafter named pMB54.

Cloning of pRR1

Construction of a chromosomal *Phns-yfp* fusion in CSH50. The procedure was identical to the construction of a chromosomal *Pdps-yfp* fusion, except Primers MBPD134/MBPD135 were used to generate a PCR product for RedE/T recombination, thereby precisely replacing the start codon of *hns* by *yfp*. The synthetic junction was amplified from chromosomal DNA by PCR, the PCR product sequenced. The resulting strain was hereafter named CSH50 P*hns-yfp*. The P*hns-yfp-cat* containing DNA fragment was PCR-amplified with Phusion DNA Polymerase and Primers MBPD102/MBPD152, KpnI digested and cloned into a SmaI/KpnI digested pUCTER backbone. The P*hns-yfp* fragment of the resulting plasmid was sequenced and the plasmid hereafter named pRR1.

Cloning of pVGfis4

Construction of a chromosomal *Pfis-yfp* fusion in CSH50. The procedure was identical to the construction of a chromosomal *Pdps-yfp* fusion, except Primers MBPD38/MBPD107 were used to generate a PCR product for RedE/T recombination, thereby precisely replacing the

start codon of *fis* by *yfp*. The synthetic junction was amplified from chromosomal DNA by PCR, the PCR product sequenced. The resulting strain was hereafter named CSH50 *Pfis-yfp*. The *Pfis-dusB-yfp-cat* containing DNA fragment was PCR-amplified with Phusion DNA Polymerase and Primers Pfisyfp/MBPD88, KpnI digested and cloned into a SmaI/KpnI digested pUCTER backbone. The *Pfis-dusB-yfp* fragment of the resulting plasmid was sequenced and the plasmid hereafter named pVGfis4.

Table S1. Primers and bacterial strains used in this study

A. Primers used in this study

Primer	Function	Sequence
c/yfp_rev	cloning	TTACTTGTACAGCTCGTCCATG
MBPD38	fisP-yfp	TTCCCCATGCCGAGTAGCGCCTTTTTAATCAAGCATTTA GCTAACCTGAAAGGAAACAGCTATGACCATG
MBPD39	OR	AGCGTCGCGTAACCCCCAGAAACATCAACGGCTCCTGA ATCAGGAGCCGTACGACGTTGTAAAACGACGG
MBPD40	OR	CTGCAAACAGAGATGGCTTAACCAAAGTGCTATGCAGT AAAAAGTGCTATAGGAAACAGCTATGACCATG
MBPD79	cloning	CCAATGCATGTTGAATACTCATACTCTTCC
MBPD80	cloning	AACTGCAGCGCCGAATAAATACCTG
MBPD81	cloning	GGGGTACCTGGCCTCAGGCATTTGA
MBPD88	cloning	AGGAAACAGCTATGACCATG
MBPD107	fisP-yfp	ATACTTCGAAAATTTTGCGTAAACAGAAATAAAGAGCT GACAGAACTATGGTGTCTATCACTAAAGATC
MBPD120	TL	CAACGTGAAATACGACTAATAACAAGCAAGACGAGCA AGTGGCTAATAATACGACGTTGTAAAACGACGG
MBPD121	TL	ATCATCGCTAAAAAAAGCCCCCTCATCATGAGGGGGA AATGCAGACACCTAGGAAACAGCTATGACCATG
MBPD132	dpsP-yfp	TTAATCTCGTTAATTACTGGGACATAACATCAAGAGGA TATGAAATTATGGTGTCTATCACTAAAGATC
MBPD133	dpsP-yfp	AGGAAGCCGCTTTTATCGGGTACTAAAGTTCTGCACCA TCAGCGATGGATAGGAAACAGCTATGACCATG
MBPD134	hnsP-yfp	ATTATTACCTCAACAAACCACCCCAATATAAGTTTGAG ATTACTACAATGGTGTCTATCACTAAAGATC
MBPD135	hnsP-yfp	AAAAAATCCCGCCGCTGGCGGGATTTTAAGCAAGTGC AATCTACAAAAGAAGGAAACAGCTATGACCATG
MBPD136	cloning	CCCAAGCTTATAAAACGAAAGGCTCAGTCG
MBPD137	cloning	CGCGGATCCTCGAGCGGCCGCTAGCCCGGGATGCATCG

		CGAAAAAGGCCATCCGTCAGGATG
MBPD144	OR	ACGCTACGGCTGAACTGTACAAATAAACCCTCATCGTC TTGTCCGACGATACGACGTTGTAAAACGACGG
MBPD145	OR	GGCATTTGCGTTTTAATGTTCGTCATTAGCGCAAAAAA AAGCCCCCGAACAGGAAACAGCTATGACCATG
MBPD147	MLup	CCTGGAAGTTAATGAAGACCGGATTGTCGCCGTCCAGT AAATGATAAAACACGACGTTGTAAAACGACGG
MBPD148	MLup	AACATCTTAAATATAGTCTTTTCCGTCTAACTTATAGAC AAAAACGAGCCAGGAAACAGCTATGACCATG
MBPD149	ML	GTTTTGCTTGGTGAATGGTGGCGTCAGAATAAAGCCTG ATAAATCAGCCGACGACGTTGTAAAACGACGG
MBPD150	ML	AGTATTACCGGCAGAGAGTGAGTAAATTTGCGGGGAA ATGCCGGATGGCAAGGAAACAGCTATGACCATG
MBPD156	cloning	GGCTGTCGCTATTCTCTCG
MBPD165	TR	ATTGAGATCCCTCGCCTGGGCCTGGCCAAATAAAAAAT CCCCGGAAGGCACGACGTTGTAAAACGACGG
MBPD166	TR	AAACAACTCCAGGCCCCGCGTCTGCGTAACTAATCCCT GAACAAATCCCCAGGAAACAGCTATGACCATG
MBPD169	MR	TTCGTAGGCCAGATAAGGCGTTCACGCCGCATCTGGCA TTTGGCTCTCGACACGACGTTGTAAAACGACGG
MBPD170	MR	CGGATGTAAAATATGAAAAATATGTAGGCATGATAAG ACGCGCCAGCGTCAGGAAACAGCTATGACCATG
Pfisyfp	cloning	GGTGGTCGCTAACATCCTTG
rplL_for	cloning	CATGCCATGGTGTCTATCACTAAAGATCAAATCGTGAG CAAGGGCGAGGA

B. Bacterial strains used in this study

**Strain**	**Genotype**	**Reference and/or construction**[a]
CSH50	*ara D(lac pro) thi rpsL*	Miller, 1972
CSH50 dpsP-*yfp*	CSH50 except *dps::yfp-cm*^{R}	RedE/T recombination
CSH50 fisP-*yfp*	CSH50 except *fis::yfp- cm*^{R}	RedE/T recombination
CSH50 hnsP-*yfp*	CSH50 except *hns::yfp- cm*^{R}	RedE/T recombination
CSH50 *dps*$^-$	CSH50 except *dps::kan*^{R}	Berger et al 2010
CSH50 *fis*$^-$	CSH50 except *fis::kan*^{R}	Koch et al 1992
CSH50 *hns*$^-$	CSH50 except *hns::tet*^{R}	Berger et al 2010
CSH50 *hupA/B*$^-$	CSH50 except *hupA::gm*^{R} *hupB::hyg*^{R}	Berger et al 2010
CSH50 dpsP-*yfp* OL	CSH50 except dpsP- *yfp-cm*^{R} in OL	RedE/T recombination
CSH50 dpsP-*yfp* OR	CSH50 except dpsP- *yfp-cm*^{R} in OR	RedE/T recombination
CSH50 dpsP-*yfp* ML	CSH50 except dpsP- *yfp-cm*^{R} in ML	RedE/T recombination
CSH50 dpsP-*yfp* MR	CSH50 except dpsP- *yfp-cm*^{R} in MR	RedE/T recombination
CSH50 dpsP-*yfp* TL	CSH50 except dpsP- *yfp-cm*^{R} in TL	RedE/T recombination
CSH50 dpsP-*yfp* TR	CSH50 except dpsP- *yfp-cm*^{R} in TR	RedE/T recombination
CSH50 dpsP-*yfp fis*$^-$	CSH50 dpsP- *yfp- cm*^{R} except *fis::kan*^{R}	P1 (CSH50 *fis::kan*^{R}) x CSH50 dpsP-*yfp*
CSH50 dpsP-*yfp* OL *fis*$^-$	CSH50 dpsP- *yfp- cm*^{R} OL except *fis::kan*^{R}	P1 (CSH50 *fis::kan*^{R}) x CSH50 dpsP-*yfp* OL
CSH50 dpsP-*yfp* OR *fis*$^-$	CSH50 dpsP- *yfp- cm*^{R} OR except *fis::kan*^{R}	P1 (CSH50 *fis::kan*^{R}) x CSH50 dpsP-*yfp* OR

CSH50 dpsP-*yfp* ML *fis*⁻	CSH50 dpsP- *yfp*- *cm*^{R} ML except *fis::kan*^{R}	P1 (CSH50 *fis::kan*^{R}) x CSH50 dpsP-*yfp* ML
CSH50 dpsP-*yfp* MR *fis*⁻	CSH50 dpsP- *yfp*- *cm*^{R} MR except *fis::kan*^{R}	P1 (CSH50 *fis::kan*^{R}) x CSH50 dpsP-*yfp* MR
CSH50 dpsP-*yfp* TL *fis*⁻	CSH50 dpsP- *yfp*- *cm*^{R} TL except *fis::kan*^{R}	P1 (CSH50 *fis::kan*^{R}) x CSH50 dpsP-*yfp* TL
CSH50 dpsP-*yfp* TR *fis*⁻	CSH50 dpsP- *yfp*- *cm*^{R} TR except *fis::kan*^{R}	P1 (CSH50 *fis::kan*^{R}) x CSH50 dpsP-*yfp* TR
CSH50 dpsP-*yfp* *hns*⁻	CSH50 dpsP- *yfp*- *cm*^{R} except *hns::tet*^{R}	P1 (CSH50 *hns::tet*^{R}) x CSH50 dpsP-*yfp*
CSH50 dpsP-*yfp* OL *hns*⁻	CSH50 dpsP- *yfp*- *cm*^{R} OL except *hns::tet*^{R}	P1 (CSH50 *hns::tet*^{R}) x CSH50 dpsP-*yfp* OL
CSH50 dpsP-*yfp* OR *hns*⁻	CSH50 dpsP- *yfp*- *cm*^{R} OR except *hns::tet*^{R}	P1 (CSH50 *hns::tet*^{R}) x CSH50 dpsP-*yfp* OR
CSH50 dpsP-*yfp* ML *hns*⁻	CSH50 dpsP- *yfp*- *cm*^{R} ML except *hns::tet*^{R}	P1 (CSH50 *hns::tet*^{R}) x CSH50 dpsP-*yfp* ML
CSH50 dpsP-*yfp* MR *hns*⁻	CSH50 dpsP- *yfp*- *cm*^{R} MR except *hns::tet*^{R}	P1 (CSH50 *hns::tet*^{R}) x CSH50 dpsP-*yfp* MR
CSH50 dpsP-*yfp* TL *hns*⁻	CSH50 dpsP- *yfp*- *cm*^{R} TL except *hns::tet*^{R}	P1 (CSH50 *hns::tet*^{R}) x CSH50 dpsP-*yfp* TL
CSH50 dpsP-*yfp* TR *hns*⁻	CSH50 dpsP- *yfp*- *cm*^{R} TR except *hns::tet*^{R}	P1 (CSH50 *hns::tet*^{R}) x CSH50 dpsP-*yfp* TR
CSH50 dpsP-*yfp* *hupA/B*⁻	CSH50 dpsP- *yfp*- *cm*^{R} except *hupA::gmR hupB::hygR*	P1 (CSH50 *hupA::gmR hupB::hygR*) x CSH50 dpsP-*yfp*
CSH50 dpsP-*yfp* OL *hupA/B*⁻	CSH50 dpsP- *yfp*- *cm*^{R} OL except *hupA::gmR hupB::hygR*	P1 (CSH50 *hupA::gmR hupB::hygR*) x CSH50 dpsP-*yfp* OL
CSH50 dpsP-*yfp* OR *hupA/B*⁻	CSH50 dpsP- *yfp*- *cm*^{R} OR except *hupA::gmR hupB::hygR*	P1 (CSH50 *hupA::gmR hupB::hygR*) x CSH50 dpsP-*yfp* OR
CSH50 dpsP-*yfp* ML *hupA/B*⁻	CSH50 dpsP- *yfp*- *cm*^{R} ML except *hupA::gmR hupB::hygR*	P1 (CSH50 *hupA::gmR hupB::hygR*) x CSH50 dpsP-*yfp* ML
CSH50 dpsP-*yfp* MR	CSH50 dpsP- *yfp*- *cm*^{R} MR	P1 (CSH50 *hupA::gmR*

hupA/B$^-$	except *hupA::gmR hupB::hygR*	*hupB::hygR*) x CSH50 dpsP-*yfp* MR
CSH50 dpsP-*yfp* TL *hupA/B*$^-$	CSH50 dpsP- *yfp*- *cm*^{R} TL except *hupA::gmR hupB::hygR*	P1 (CSH50 *hupA::gmR hupB::hygR*) x CSH50 dpsP-*yfp* TL
CSH50 dpsP-*yfp* TR *hupA/B*$^-$	CSH50 dpsP- *yfp*- *cm*^{R} TR except *hupA::gmR hupB::hygR*	P1 (CSH50 *hupA::gmR hupB::hygR*) x CSH50 dpsP-*yfp* TR
CSH50 fisP-*yfp* MLup	CSH50 except fisP- *yfp*- *cm*^{R} in MLup	RedE/T recombination
CSH50 fisP-*yfp* ML	CSH50 except fisP- *yfp*- *cm*^{R} in ML	RedE/T recombination
CSH50 fisP-*yfp* MLup *hupA/B*$^-$	CSH50 fisP- *yfp*- *cm*^{R} in MLup except *hupA::gmR hupB::hygR*	P1 (CSH50 *hupA::gmR hupB::hygR*) x CSH50 fisP-*yfp* MLup
CSH50 fisP-*yfp* ML *hupA/B*$^-$	CSH50 fisP- *yfp*- *cm*^{R} in ML except *hupA::gmR hupB::hygR*	P1 (CSH50 *hupA::gmR hupB::hygR*) x CSH50 fisP-*yfp* ML
DH5α	*F' / endA1 hsdR17 (rk*$^-$ *mk*$^+$*) glnV44 thi-1 recA1 gyrA (nal*^{R}*) relA1 D(lacIZYA-argF)U169 deoR (j80dlacD(lacZ)M15)*	invitrogen™

[a]Constructions are shown as P1 (donor) x recipient ; markers were transduced subsequently when necessary.

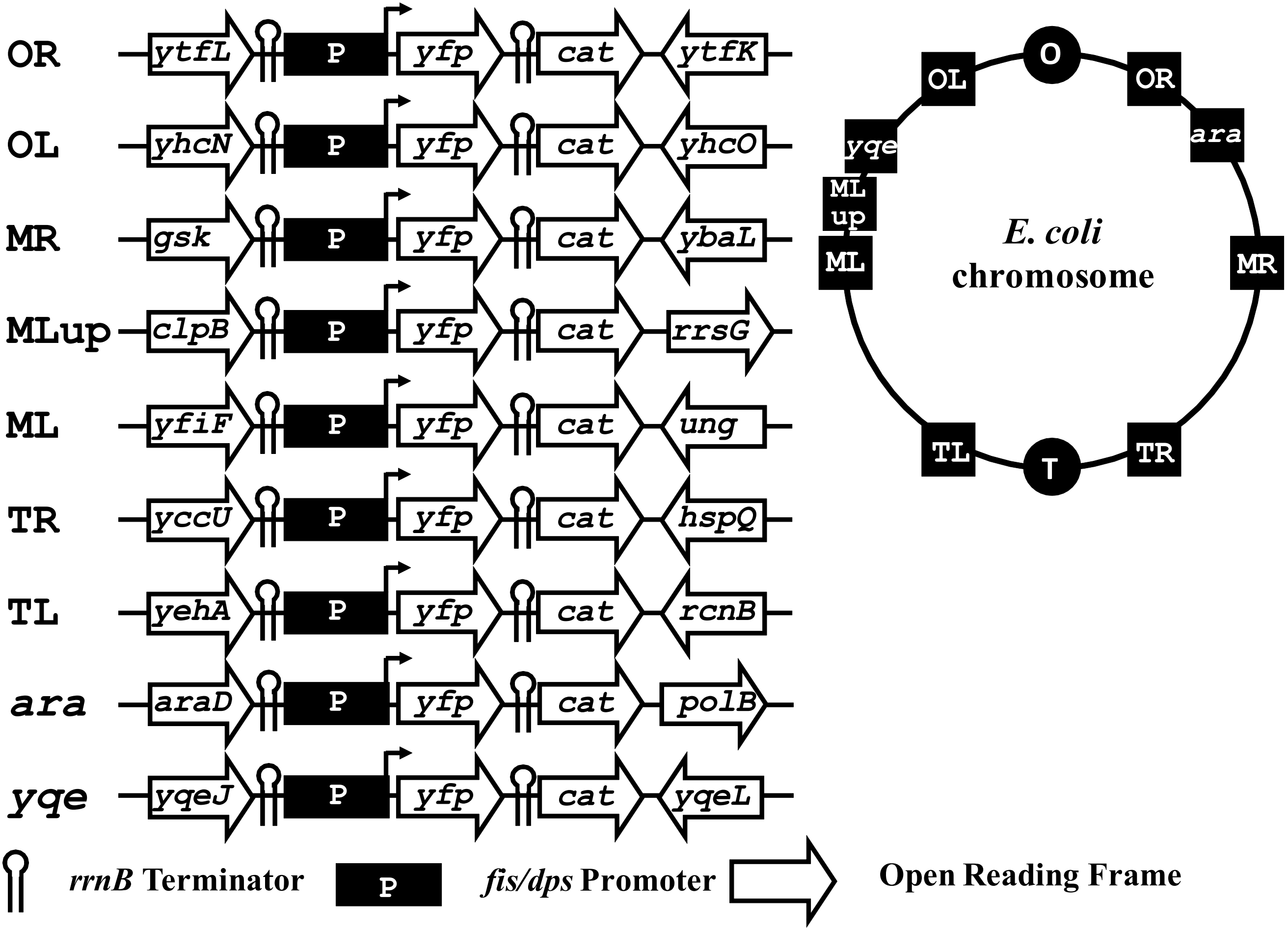

Figure S1. Graphic representation of the exact position of the modules. Shown are the modules and the neighboring ORFs, as well as the approximate position of the modules in the *E. coli* chromosome.

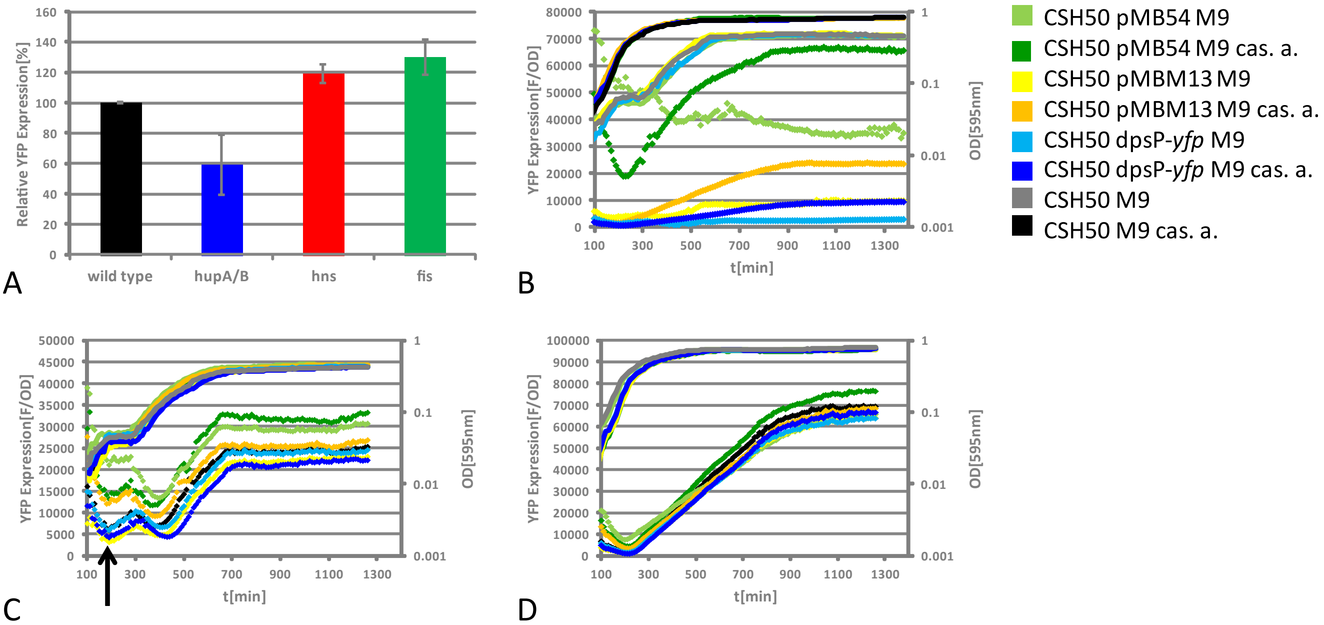

Figure S2. Technical analysis of the P*dps* module. (A) Comparison of P*dps* module (pMB54) dependent YFP expression in *hupA/B*, *hns* and *fis* mutants to wild type (= 100%). Shown are average values from endpoint measurements of kinetic measurements and standard deviations of two independent biological replicates. (B) P*dps* module dependent YFP expression from a high copy plasmid (pMB54), a low copy plasmid (pMBM13) and from the original chromosomal locus of *dps* (CSH50 dpsP-*yfp*) in M9 and M9 supplemented with casamino acids.The increased YFP expression from the plasmids does not affect the growth characteristics (compare coloured lines to black line (CSH50 M9 cas.a.) and grey line (CSH50 M9). Note that the lower expression values of CSH50 dpsP-*yfp* when compared to C and D are resulting from the reduced detector gain of the instrument in this experiment. The colour code is depicted next to the graph. (C) P*dps* module dependent YFP expression in M9; black arrow indicates activation of the dps promoter at the intermediate lag phase. (D) P*dps* module dependent YFP expression in M9 supplemented with casamino acids. The P*dps* module produces more YFP when M9 is supplemented with casamino acids (compare diamonds in C and D). The YFP expression pattern of the original *dps*P-*yfp* fusion is similar to that of the transplaced modules (compare black diamonds to coloured diamonds in C and D). The transplaced modules do not affect the growth characteristics of the strains (compare grey line to coloured lines) Grey line: CSH50; black line CSH50 dpsP-*yfp*; black diamonds: YFP expression from CSH50 dpsP-*yfp*; colour code for the rest of the modules: See figure 2.

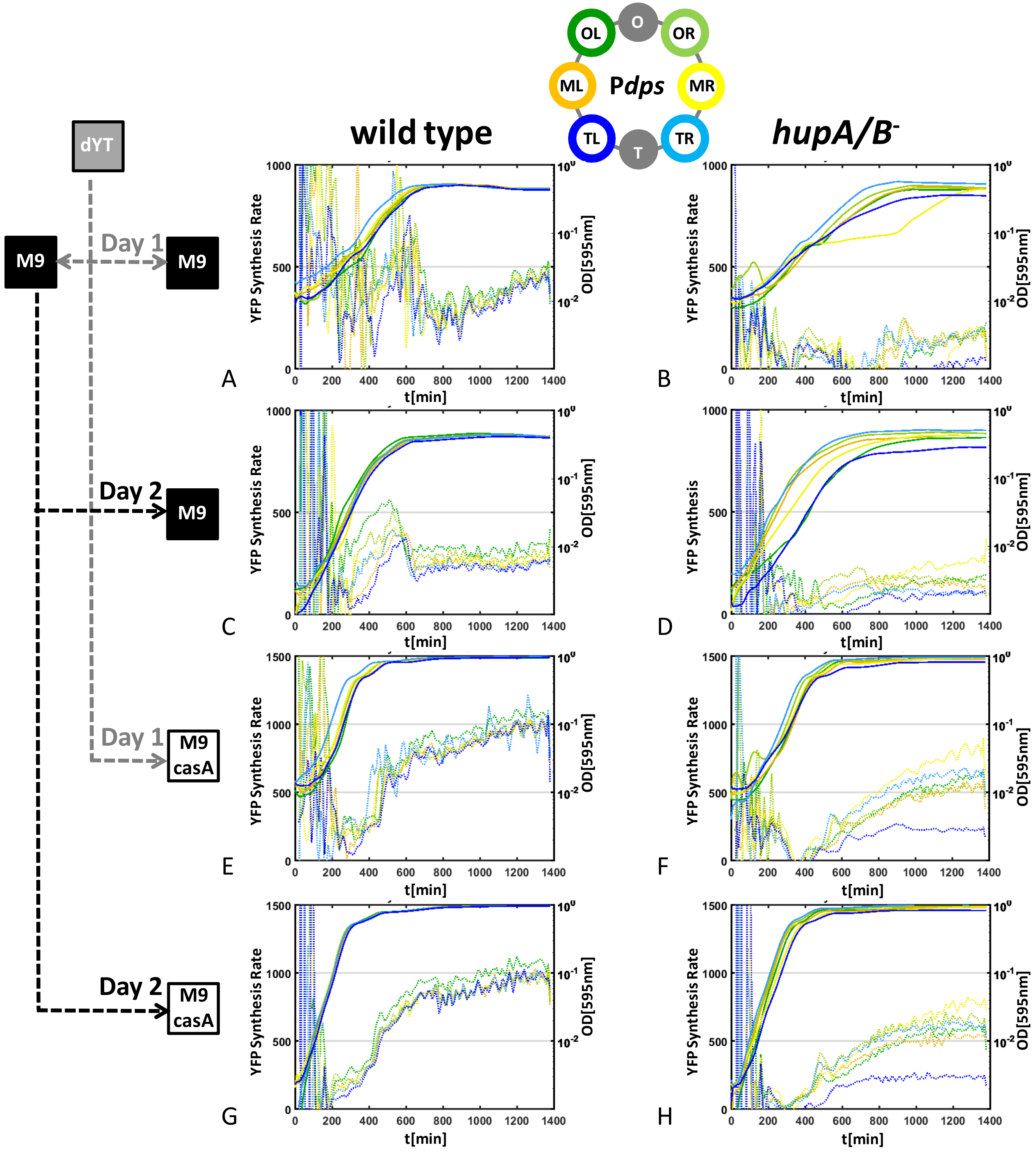

Figure S3. P*dps* module dependent YFP synthesis rates in wild type and *hupA/B* mutant. (A-H) The data shown in Figure 2 was normalized according to De Jong et al., 2010 to calculate the YFP synthesis rates of the modules (dots) and the growth curves (lines). The accumulation of YFP shown in Figure 2 correlates with the YFP synthesis rates of the individual P*dps* modules. For details see text.

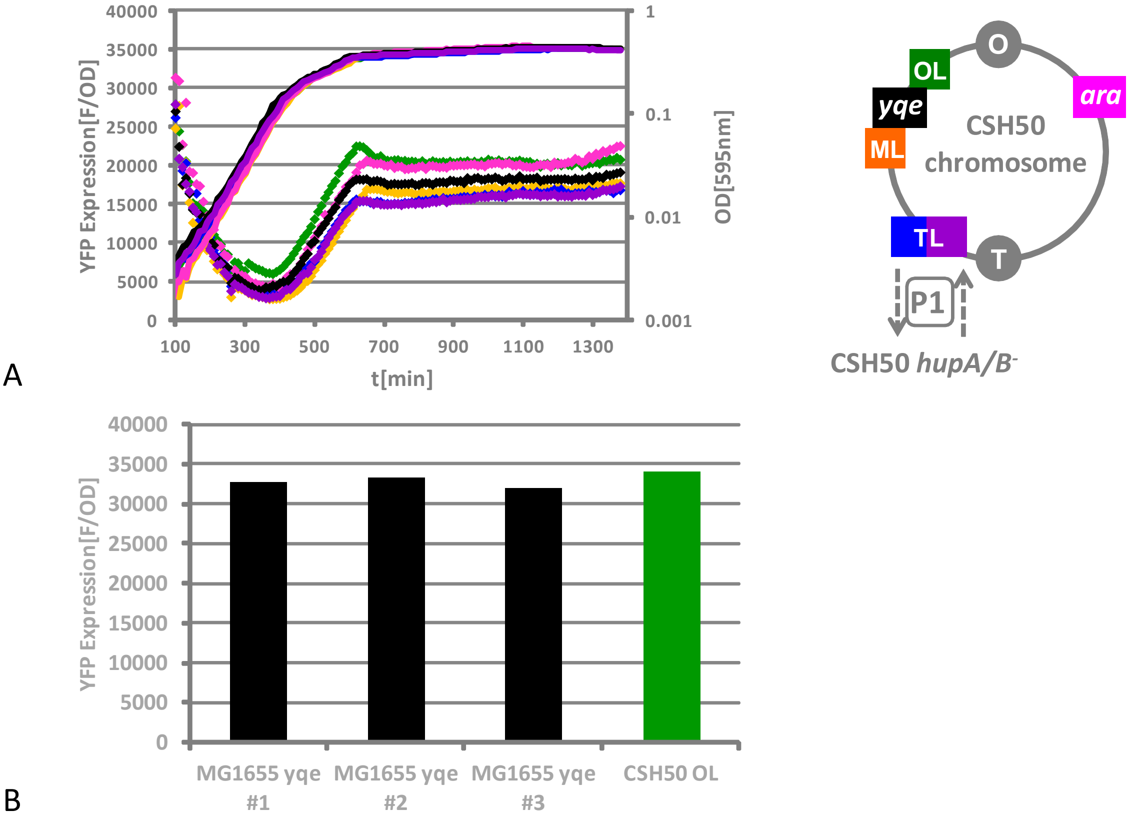

Figure S4. Technical tests. (A) YFP expression (diamonds) and growth curves (lines) of CSH50 wild type cells with P*dps* module insertions in *yqe*, *ara* and the re-transductand of the P*dps* module of CSH50 *hupA/B-* TL into CSH50, as well as the modules OL, ML and TL in M9 medium supplemented with glucose. Except for a slight copy number effect, the P*dps* module is not affected by the chromosomal position in wild type cells. Colour code and approximate position of the modules are graphically depicted next to the graph. (B)YFP expression in dYT overnight cultures. The P*dps* module *yqe* was transduced into *E. coli* MG1655 and three colonies were inoculated in dYT medium. YFP production in *E. coli* MG1655 is comparable to *E. coli* CSH50, excluding the possibility that the normal expression of the P*dps* module *yqe* is specific for the genetic background (compare black bars with green bar).

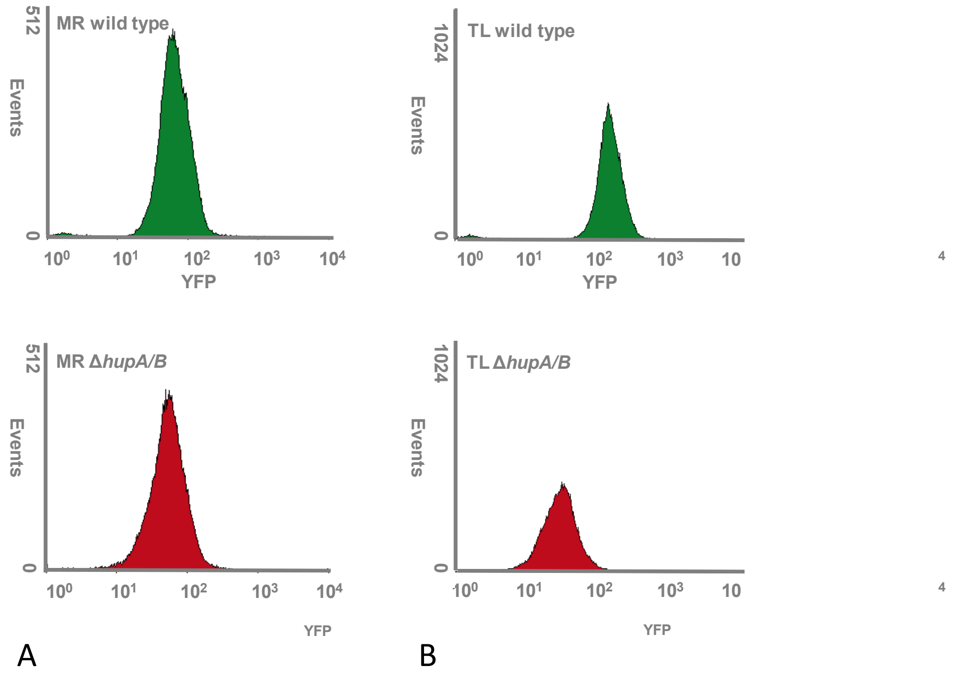

Figure S5. Analysis of YFP signal distribution within bacterial populations by FACS. (A) Signal distribution in wild type (top) and *hupA/B* mutants (bottom) when the P*dps* module is localized in MR. (B) Signal distribution in wild type (top) and *hupA/B* mutants (bottom) when the P*dps* module is localized in TL. As for wild type cells, the signals in *hupA/B* mutant cells are normally distributed, i.e. the lower YFP signal produced by the mutants is not resulting from a heterogeneous population with respect to YFP expression.

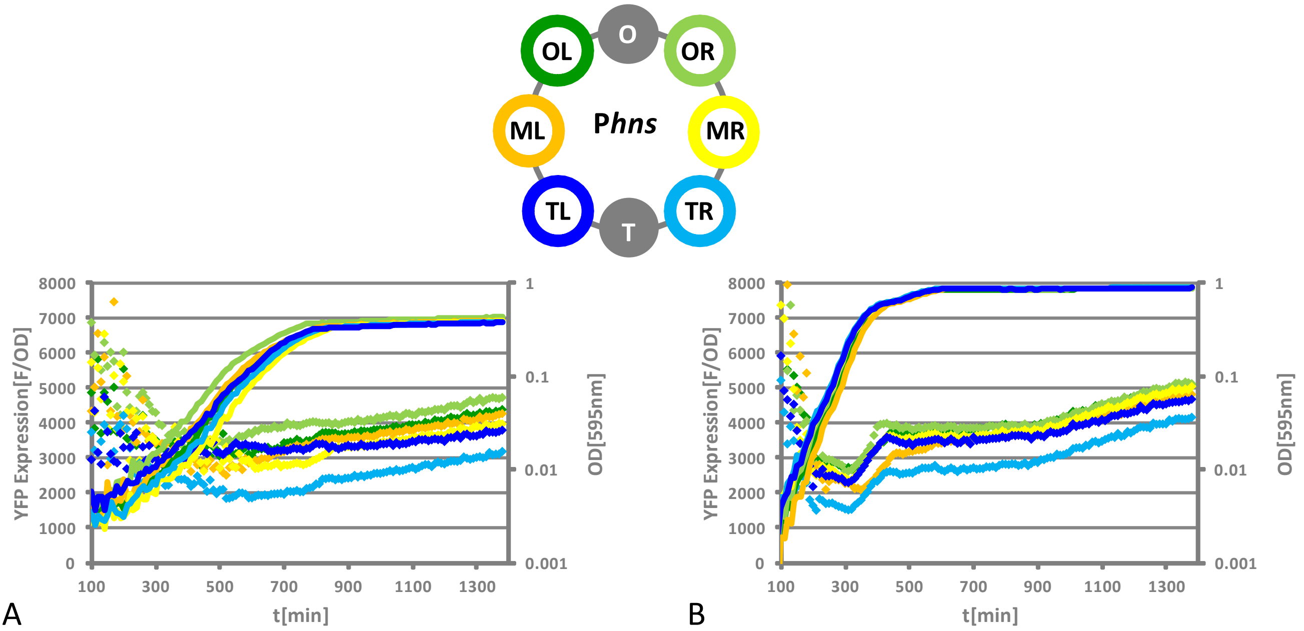

Figure S6. P*hns* module dependent YFP expression in *hns* mutants in M9 (A) and M9 supplemented with casamino acids (B). The lower YFP expression values of the P*hns* module TL are an H-NS dependent, promoter-chromosomal backbone fusion specific artefact (compare diamonds to wild type, Figure 4).

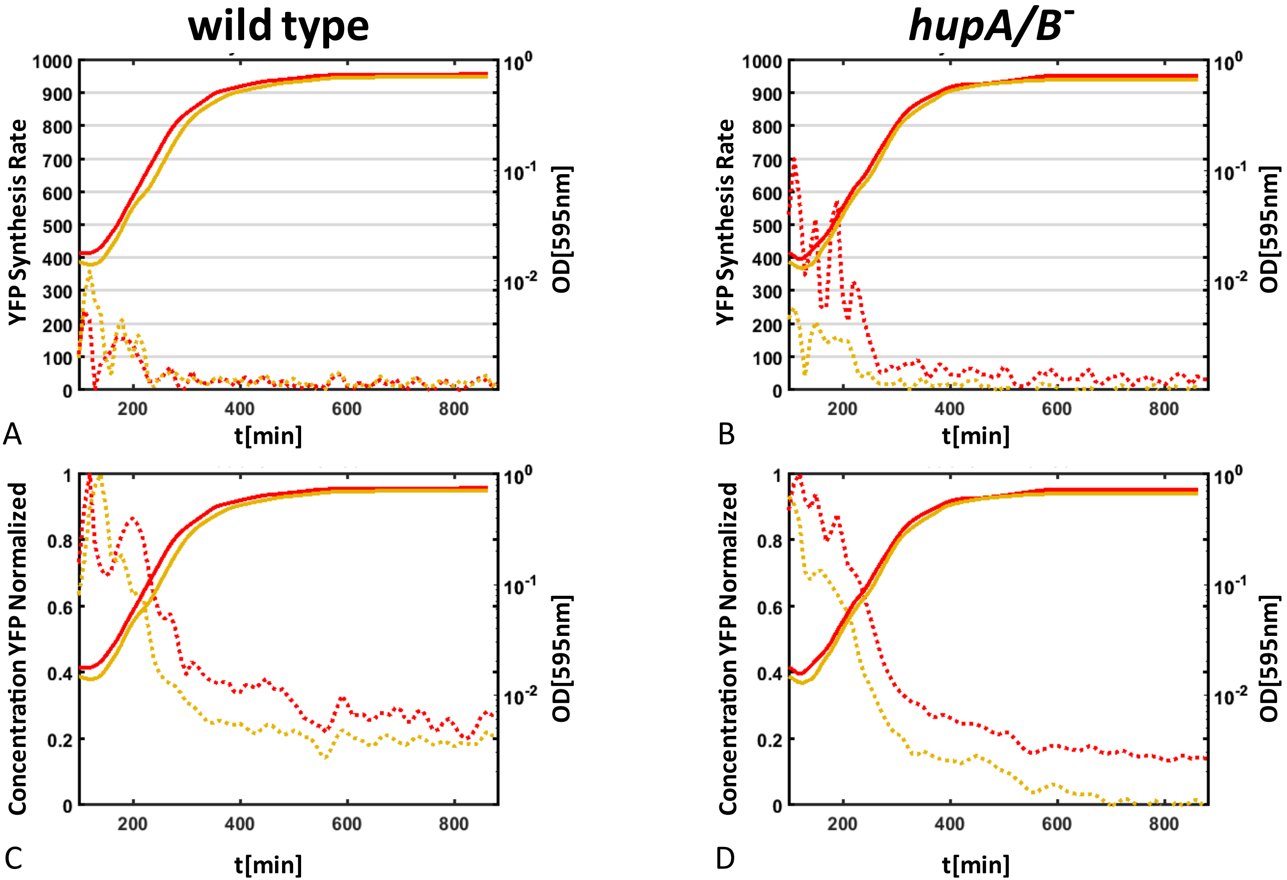

Figure S7. YFP synthesis rate and normalized YFP concentrations of the *fis* promoter modules in wild type (A and C) and *hupA/B* (B and D) mutant. The YFP synthesis rate of module Mlup is increased when compared to module ML in *hupA/B* mutant, but not in wild type cells (compare red and orange dots in A and B). The overall promoter activity pattern is similar for both modules (compare red and orange dots in C and D). The color code is identical to Figure 5.

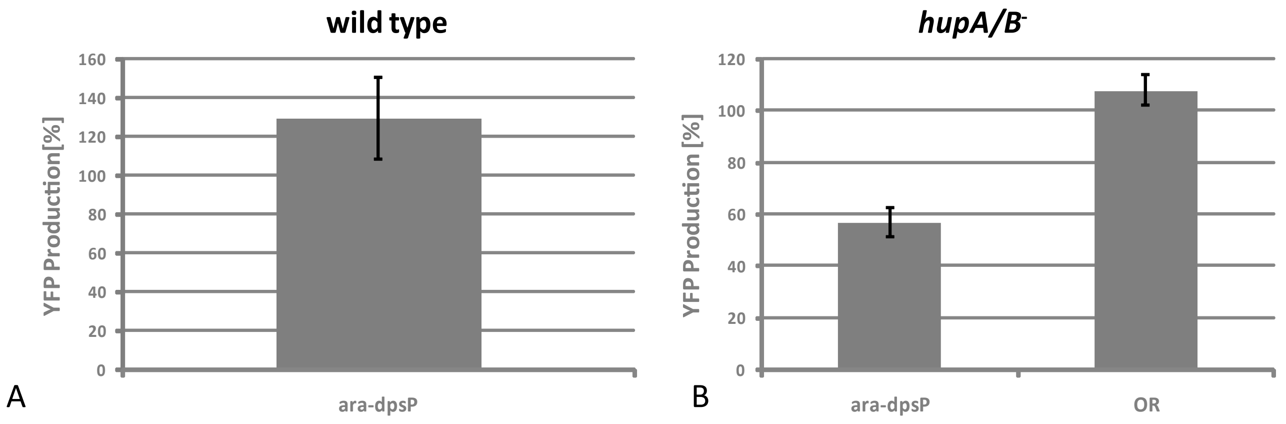

Figure S8. Module proximal transcription and promoter activity. (A) The P*dps* modul *ara* produces slightly more YFP when wild type cells are grown in Arabinose than when grown in Glucose. (B) In contrast to the P*dps* module OR, the P*dps* module *ara* produces less YFP when the *hupA/B* mutants are grown in Arabinose than when grown in Glucose. Shown are average values and standard deviations from three independent experiments.

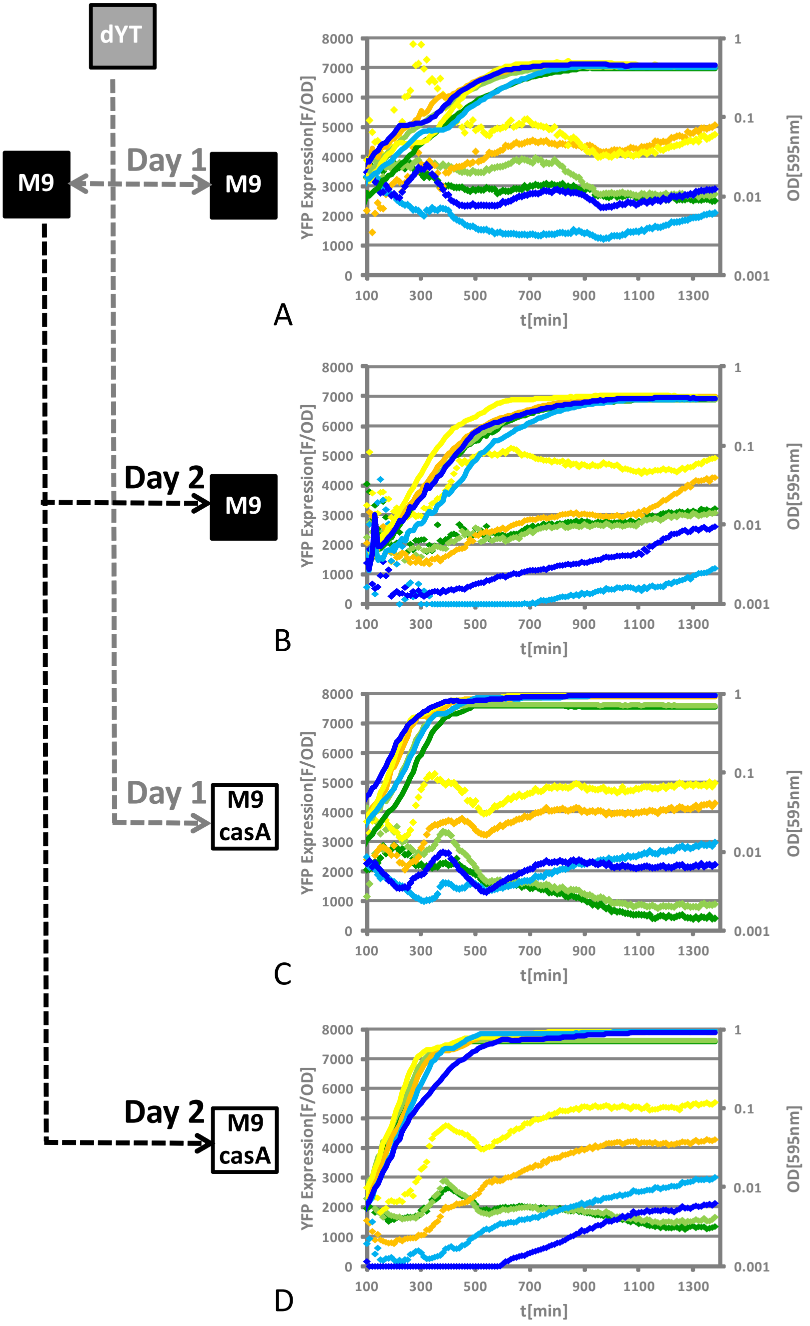

Figure S9. Lack of P*hns* module stability in certain positions in the *hupA/B* mutant. Shown is another biological replicate of the experiment shown in figure 4. The P*hns* module dependent YFP expression patterns are stable within, but not all are stable in between biological replicates in the *hupA/B* mutant (compare e.g. dark green diamonds with Figure 4). For colour code see Figure 4.

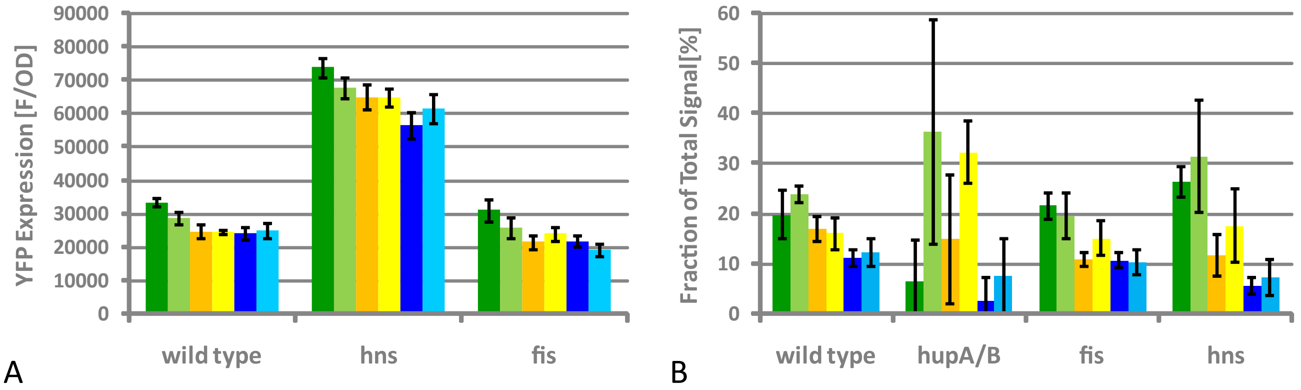

Figure S10. P*dps* module activity in different genetic backgrounds. (A) Final P*dps* module dependent YFP production in wild type, *hns* and *fis* mutant in M9 medium. The P*dps* modules produce approximately 2-fold more YFP in *hns* mutants than in wild type, but do not show position dependence. Shown are average values and standard deviations of the final YFP production (stationary phase) of 5 biological replicates. (B) P*dps* module response to hydrogen peroxide challenge in wild type and NAP mutants as fraction of total signal produced by all six modules (Expected value if all modules contribute equally 100/6 ~ 17%). Shown are average values and standard deviations of 5 biological replicates.
